# Multimodal evaluation of hypoxia in brain metastases of lung cancer and interest of hypoxia image-guided radiotherapy

**DOI:** 10.1038/s41598-021-90662-0

**Published:** 2021-05-27

**Authors:** Aurélien Corroyer-Dulmont, Samuel Valable, Jade Fantin, Laurent Chatre, Jérôme Toutain, Sylvain Teulier, Céline Bazille, Elise Letissier, Jérôme Levallet, Didier Divoux, Méziane Ibazizène, Stéphane Guillouet, Cécile Perrio, Louisa Barré, Sébastien Serres, Nicola R. Sibson, Françoise Chapon, Guénaëlle Levallet, Myriam Bernaudin

**Affiliations:** 1 Normandie Univ, UNICAEN, CEA, CNRS, ISTCT/CERVOxy Group, GIP CYCERON, 14000 Caen, France; 2 Medical Physics Department, CLCC François Baclesse, 14000 Caen, France; 3 Normandie Univ, UNICAEN, CEA, CNRS, ISTCT/LDM-TEP Group, GIP CYCERON, 14000 Caen, France; 4 Medical Research Council Oxford Institute for Radiation Oncology, Department of Oncology, University of Oxford, Oxford, UK; 5 School of Life Sciences, University of Nottingham, Nottingham, UK; 6 Department of Pathology, University Hospital of Caen, Caen, France; 7 Department of Pulmonology and Thoracic Oncology, University Hospital of Caen, Caen, France

**Keywords:** Cancer microenvironment, CNS cancer, Metastasis, Cancer therapy, Radiotherapy

## Abstract

Lung cancer patients frequently develop brain metastases (BM). Despite aggressive treatment including neurosurgery and external-radiotherapy, overall survival remains poor. There is a pressing need to further characterize factors in the microenvironment of BM that may confer resistance to radiotherapy (RT), such as hypoxia. Here, hypoxia was first evaluated in 28 biopsies from patients with non‑small cell lung cancer (NSCLC) BM, using CA-IX immunostaining. Hypoxia characterization (pimonidazole, CA-IX and HIF-1α) was also performed in different preclinical NSCLC BM models induced either by intracerebral injection of tumor cells (H2030-Br3M, H1915) into the cortex and striatum, or intracardial injection of tumor cells (H2030-Br3M). Additionally, [^18^F]-FMISO-PET and oxygen-saturation-mapping-MRI (SatO2-MRI) were carried out in the intracerebral BM models to further characterize tumor hypoxia and evaluate the potential of Hypoxia-image-guided-RT (HIGRT). The effect of RT on proliferation of BM ([^18^F]-FLT-PET), tumor volume and overall survival was determined. We showed that hypoxia is a major yet heterogeneous feature of BM from lung cancer both preclinically and clinically. HIGRT, based on hypoxia heterogeneity observed between cortical and striatal metastases in the intracerebrally induced models, showed significant potential for tumor control and animal survival. These results collectively highlight hypoxia as a hallmark of BM from lung cancer and the value of HIGRT in better controlling tumor growth.

## Introduction

Brain tumors are most frequently brain metastases (BM), occurring 3–10 times more than primary brain tumors^1^, with 60% of cases arising from primary lung and breast cancer (40% and 20%, respectively^2^). The incidence of BM is increasing^3^. Standard treatment for multiple BM remains surgical resection, if possible combined with brain radio-therapy (RT) and/or chemotherapy^4^. RT, including whole brain radiotherapy (WBRT) and stereotactic radio-surgery (SRS), is considered the main approach for the treatment of BM from solid tumors. Despite this aggressive treatment, the median survival for patients with BM remains poor (6–9 months from diagnosis^5^) and long-term survivors frequently experience cognitive decline^6^. There is, therefore, a pressing unmet therapeutic need for better tumor control and reduction in RT dose deposition in healthy tissue. This goal could be realizable with better characterization of specific features of the tumor microenvironment that may confer in radioresistance, such as the presence of hypoxia^7^. Such characterization would allow the development of more personalized RT. Paradoxically, the literature on hypoxia in BM is still limited: Berghoff and collaborators showed the presence of hypoxia in BM using hypoxia-inducible-factor-1α (HIF-1α) immunostaining of human biopsies from lung, breast, renal and collorectal cancers^8^. These authors also showed in non-small cell lung cancer (NSCLC) that the expression of HIF-1α is a prognostic factor^9^ supporting the need to adapt RT to hypoxia in BM. More recently, Ebright and colleagues have shown, in preclinical models of BM from breast cancer, that hypoxic signaling with HIF-1α is highly correlated to tumor proliferation, and that high BM HIF-1α expression in patients is associated with a significant decrease in overall survival in comparison to the low hypoxic group^10^. Finally, target genes of hypoxia, such as *VEGF* and *β3-tubulin,* have been shown to be expressed in BM from breast cancer in both preclinical and clinical situations^11–13^. However, with the potential heterogeneity of the tumor microenvironment in BM, and therapeutic response, more personalized approaches are required. We and others have previously demonstrated, for primary brain tumors (glioblastoma-IDH1/2-Wild-Type), the potential value of multimodal imaging to characterize hypoxia which could lead to the adaptation of RT and improved tumor control^14–16^. In addition, hypoxia image-guided radiotherapy (HIGRT) would enable increasing dose deposition to radio-resistant hypoxic tumors, whilst decreasing dose to non-hypoxic radio-sensitive tumors, thus maintaining acceptable toxicity whilst increasing efficacy. HIGRT for well-known hypoxic primary tumors, such as head and neck tumors, has shown significantly higher tumor control probability^17^.

**Figure 1:**
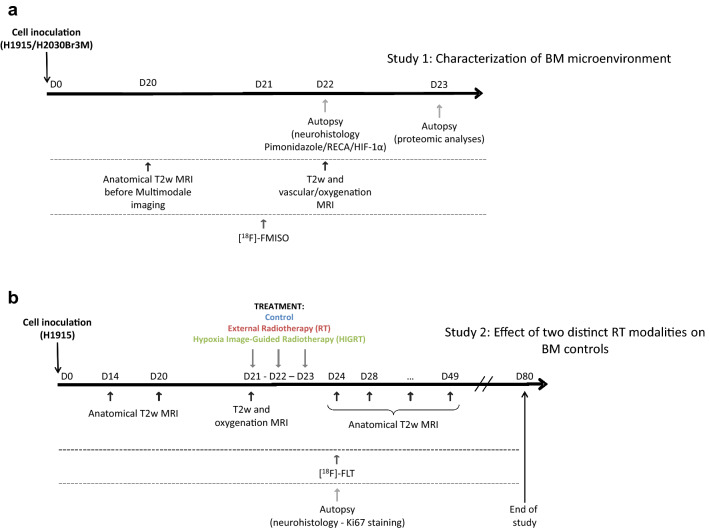
Experimental paradigm and schema for (**a**) hypoxia microenvironment characterization study and (**b**) therapy study.

The main goal of this study, was firstly to characterize the hypoxic microenvironment of BM from lung cancers, using both preclinical and clinical approaches. We focused on BM from NSCLC, since 7.4% of NSCLC patients have BM at first presentation^18^, and 25–30% develop BM during their disease course^19^. The second aim of this study was to assess, at the preclinical level, the presence or not of hypoxia in different BM models and the ability of multimodal imaging to characterize hypoxia heterogeneity in BM and to, ultimately, determine the therapeutic potential of a personalized RT based on tumor hypoxia. To achieve these aims, the hypoxic microenvironment of BM was firstly characterized in clinical biopsies from NSCLC, followed by two preclinical studies (Fig. 1a,b): (a) the microenvironment characterization of BM using immunohistochemistry and multimodal imaging, and (b) a therapeutic efficacy study for HIGRT in BM, including comparison to external beam radiotherapy (RT).

## Results

### Characterization of hypoxia in human BM biopsies from primary lung cancer by immunohistochemistry

**Figure 2:**
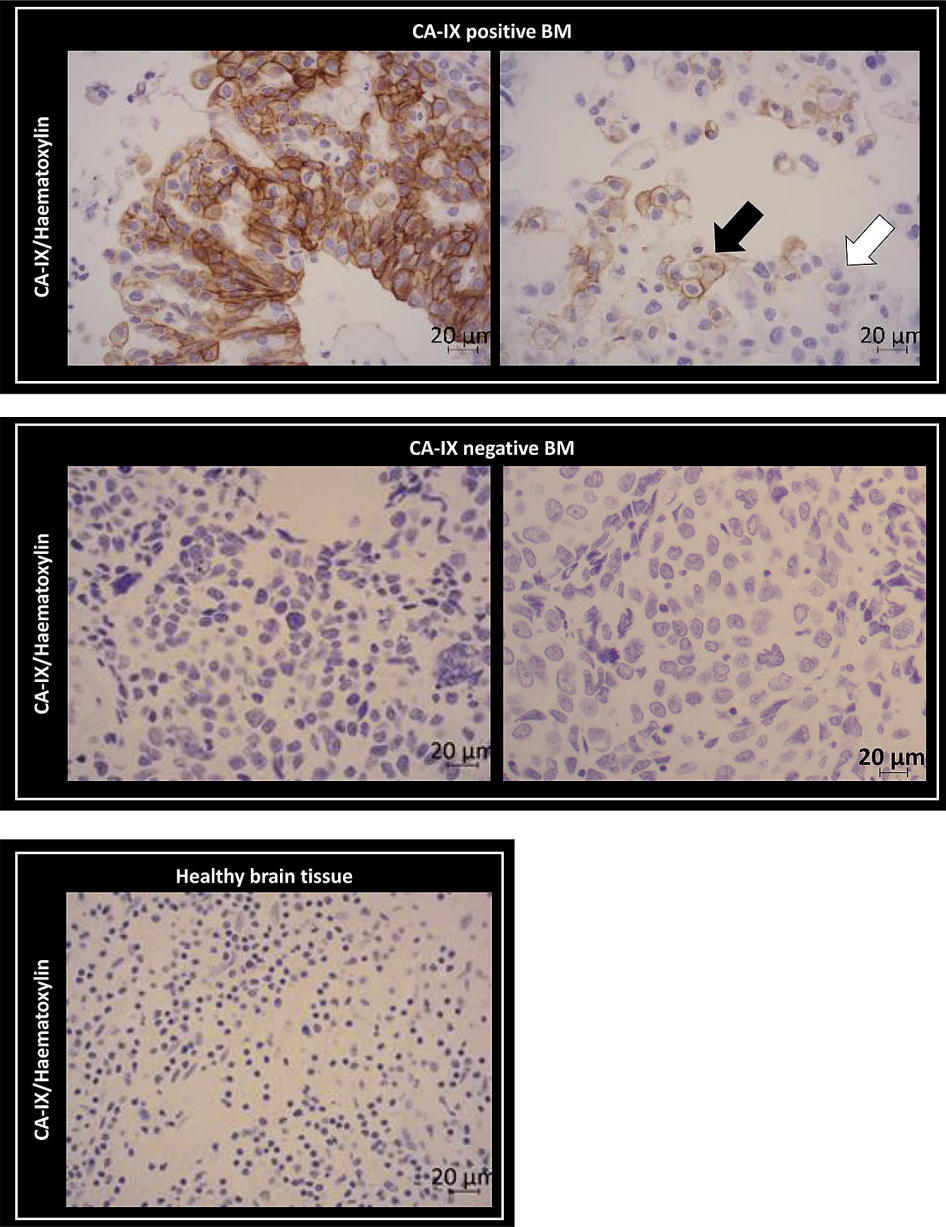
Characterization of hypoxia in human BM biopsies from primary lung cancer by immunohistochemistry. Representative images of CA-IX staining (brown) with hematoxylin counterstaining (purple) on four BM biopsies from four patients with primary lung adenocarcinoma and one healthy tissue.

We used, carbonic anhydrase-IX (CA-IX), a hypoxia-inducible factor (HIF) target widely used in the clinic as an endogenous marker of hypoxia in solid tumors^20^ and HIF-1α itself. CA-IX staining on BM from primary lung NSCLC cancer biopsies indicated that hypoxia was detected in 22 out of 28 (i.e. 78.6%) patients analyzed (Fig. 2). These results also revealed heterogeneity both between patients and within a BM (Fig. 2, black (CA-IX positive tumor cells) and white (CA-IX negative tumor cells) arrows). Results from additional staining for 4 biopsies show that a clear HIF-1α staining is observed where that of CA-IX is strong, and HIF-1α staining is not above detection levels where CA-IX is weak (Sup Fig. 1).

### Characterization of hypoxia in murine BM models from lung cancer

**Figure 3:**
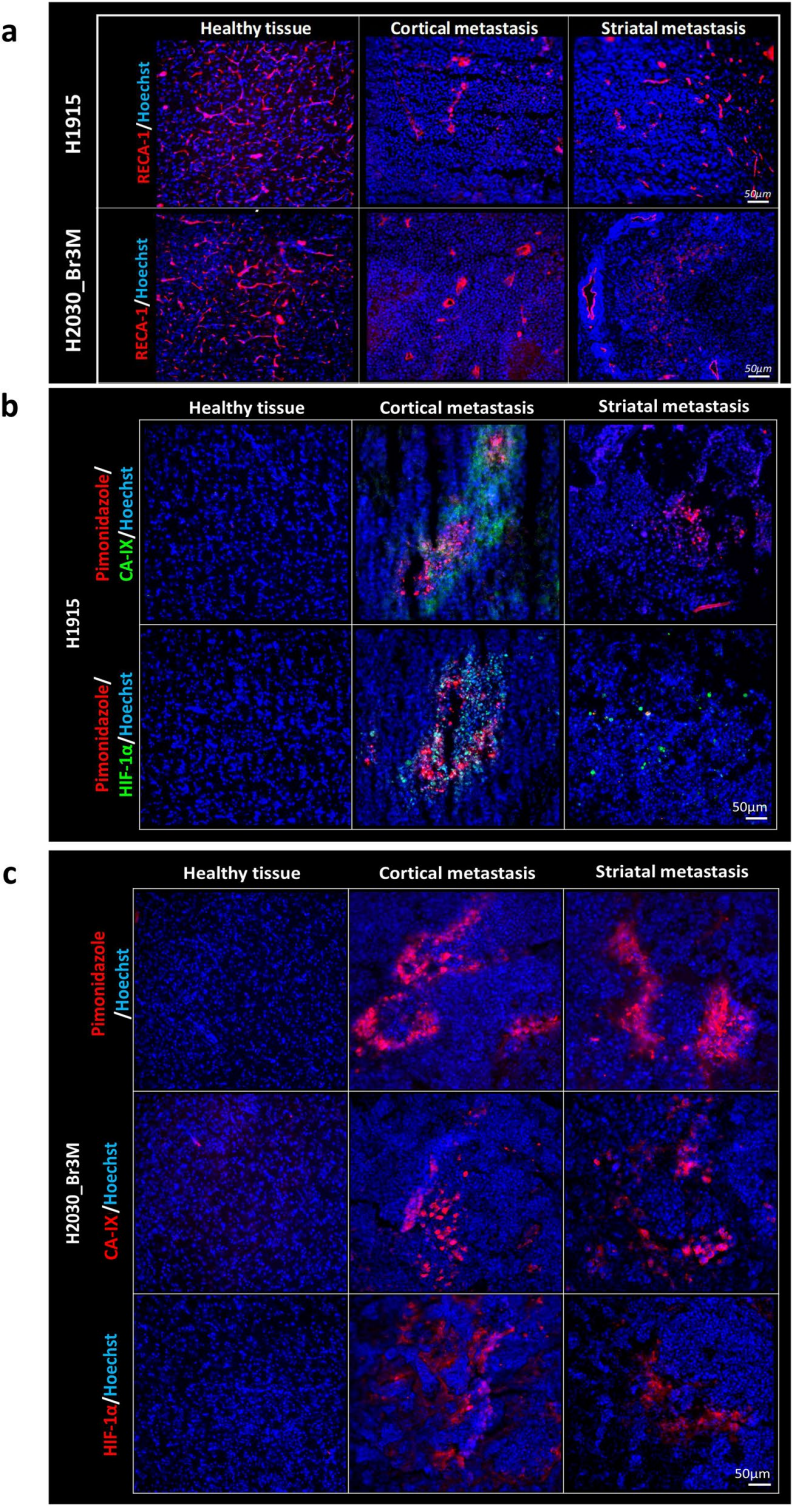
Immunohistochemical studies of vascularization and hypoxia in BM. (**a**) Representative images of RECA immunostaining (vascularization**,** red) with a Hoechst 33342 nuclear counterstaining (blue) for H1915 and H2030-Br3M models. (**b**) Representative images of pimonidazole staining (red), CA-IX staining (green, top row) and HIF-1α staining (green, bottom row) with a Hoechst 33,342 nuclear counterstain (blue) in the BM model from human lung adenocarcinoma (H1915, cortical and striatal metastasis) and healthy tissues. (**c**) Representative images of pimonidazole staining (red-top row), CA-IX staining (red-middle row) and HIF-1α (red-bottom row) with a Hoechst 33342 nuclear counterstain (blue) on BM model from human lung adenocarcinoma (H2030-Br3M, cortical and striatal metastasis) and healthy tissues.

To investigate the hypoxic feature of BM and its potential spatial heterogeneity, metastatic adenocarcinoma H2030-Br3M or H1915 cancer cells were injected into the brain of nude rats in two different regions, striatum and cortex. As hypoxia can be caused by low vascularization RECA-1 staining was firstly analyzed and showed a significant decrease in vessel density and an increase in vessel diameter and domains for both cortical and striatal metastases in comparison to healthy tissue for both H2030-Br3M and H1915 models (Fig. 3a). Interestingly, inter-BM heterogeneity was observed in the H1915 model with less vascularization in cortical BM in comparison to the striatal BM (*p* < 0.05, Table 1). Moreover, intra-BM heterogeneity was observed with a greater vascularization evident in the shell in comparison to the core of the tumor for both H2030-Br3M and H1915 models and both tumor locations. As a consequence of the low vascularization, hypoxia was expected to be strongly present. Indeed, pimonidazole, CA-IX and HIF-1α staining revealed marked staining in both models and regions (Fig. 3b,c). Interestingly, as shown in Fig. 3b, CA-IX and HIF-1α staining was more pronounced in cortical metastases than striatal metastasis in the H1915 model.

**Table 1:** Quantification of the RECA-1 immunostaining.

	Healthy tissue	Cortical metastasis, core	Cortical metastasis, shell	Striatal metastasis, core	Striatal metastasis, shell
**H2030_Br3M**					
Vessel density (vessels/mm^2^)	306.40 (27.61)	28.12 (12.41)*	50.86 (6.66)***	35.21 (44.79)***	84.12 (4.17)***
Vessel diameter (μm)	1.68 (0.19)	3.40 (0.89)	2.35 (0.22)^$^	4.69 (1.55)*	2.35 (0.17)^$^
Domains (μm)	8.02 (0.19)	49.61 (2.29)*	23.07 (3.20)	51.25 (26.77)*	14.93 (2.54)^#^/^$^
**H1915**					
Vessel density (vessels/mm^2^)	295.79 (25.03)	23.89 (17.66)***	64.56 (2.16)***	58.10 (39.92)^#^/***	68.41 (12.10)^#^/***
Vessel diameter (μm)	2.53 (0.15)	9.53 (3.32)***	2.07 (0.13)^###^	3.90 (1.78)^#^	2.45 (0.26)^###^
Domains (μm)	12.53 (2.96)	55.60 (10.54)***	33.14 (2.94)^###^/**	37.75 (4.33)^##^/***	25.89 (5.11)^###^

Data represent the mean (SD) of vessels density and diameter and domains analyzed with histology for H2030-Br3M and H1915. Mean ± SD, n = 3, *p < 0.05, **p < 0.01 and ***p < 0.001 versus healthy tissue, ^#^*p* < 0.05 and ^###^*p* < 0.001 versus cortical metastasis core and ^$^*p* < 0.05 versus striatal metastasis core.

**Figure 4:**
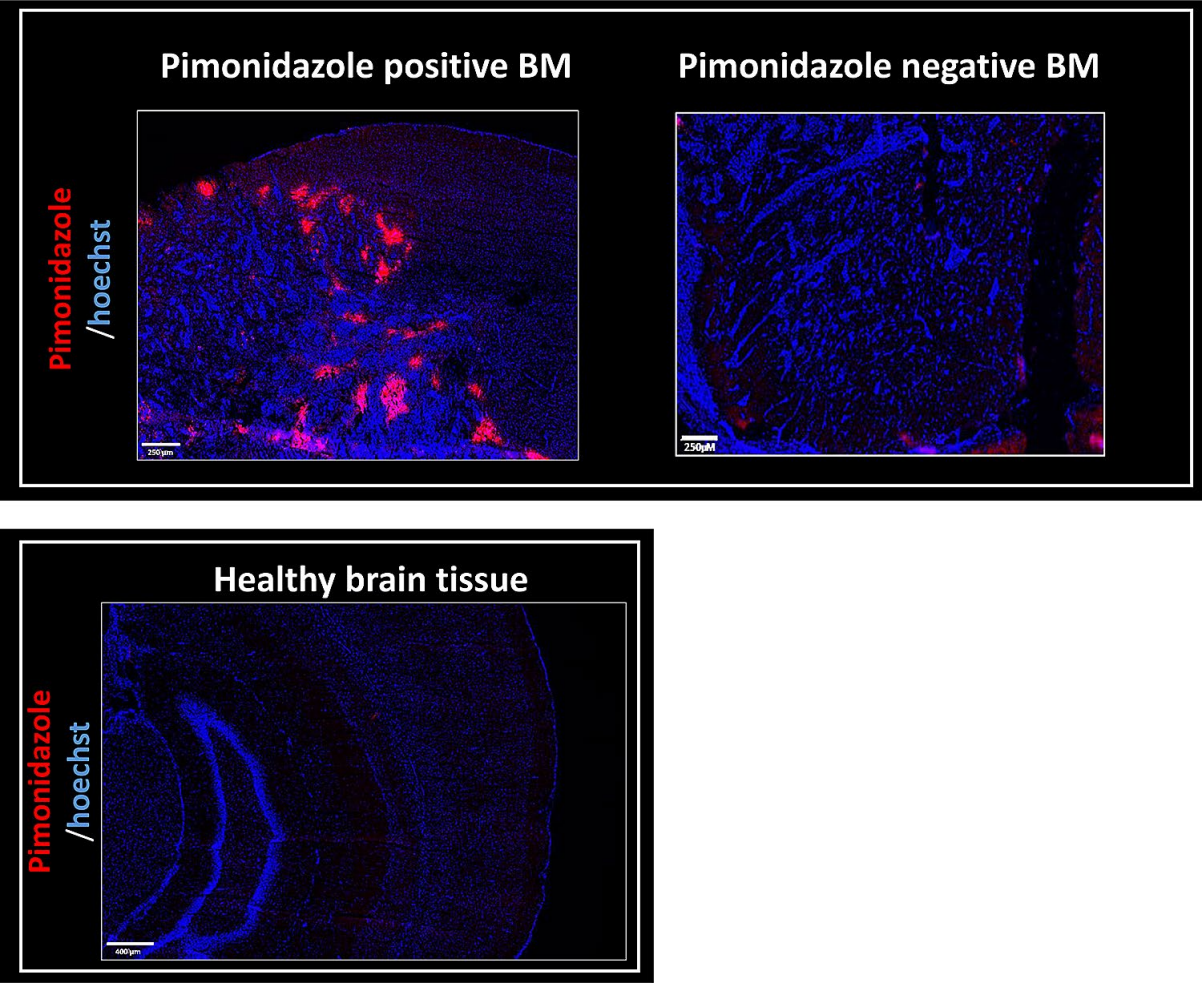
Pimonidazole detection by immunohistology in preclinical models of BM with intracardiac injection of human lung cancer (H2030-Br3M). Representative images of pimonidazole staining (red) with a Hoechst 33342 nuclear counterstaining (blue) on BM model from human lung adenocarcinoma (H2030-Br3M) and one healthy tissue.

The hypoxia feature of BM was further confirmed through additional studies in a BM model induced by intracardial injection of H2030-Br3M cells in nude rats. This model is complementary to the intracerebral model of BM since it mimics the natural route of brain parenchyma invasion. Sixty percent of the animals presented with cortical BM and twenty percent with cerebellar BM. All cortical and one of two cerebellar BM were positive for pimonidazole staining (86% of positives pimonidazole BM). These results are in line with the clinical results concerning the hypoxic status in BM from lung cancers (Fig. 4).

**Figure 5:**
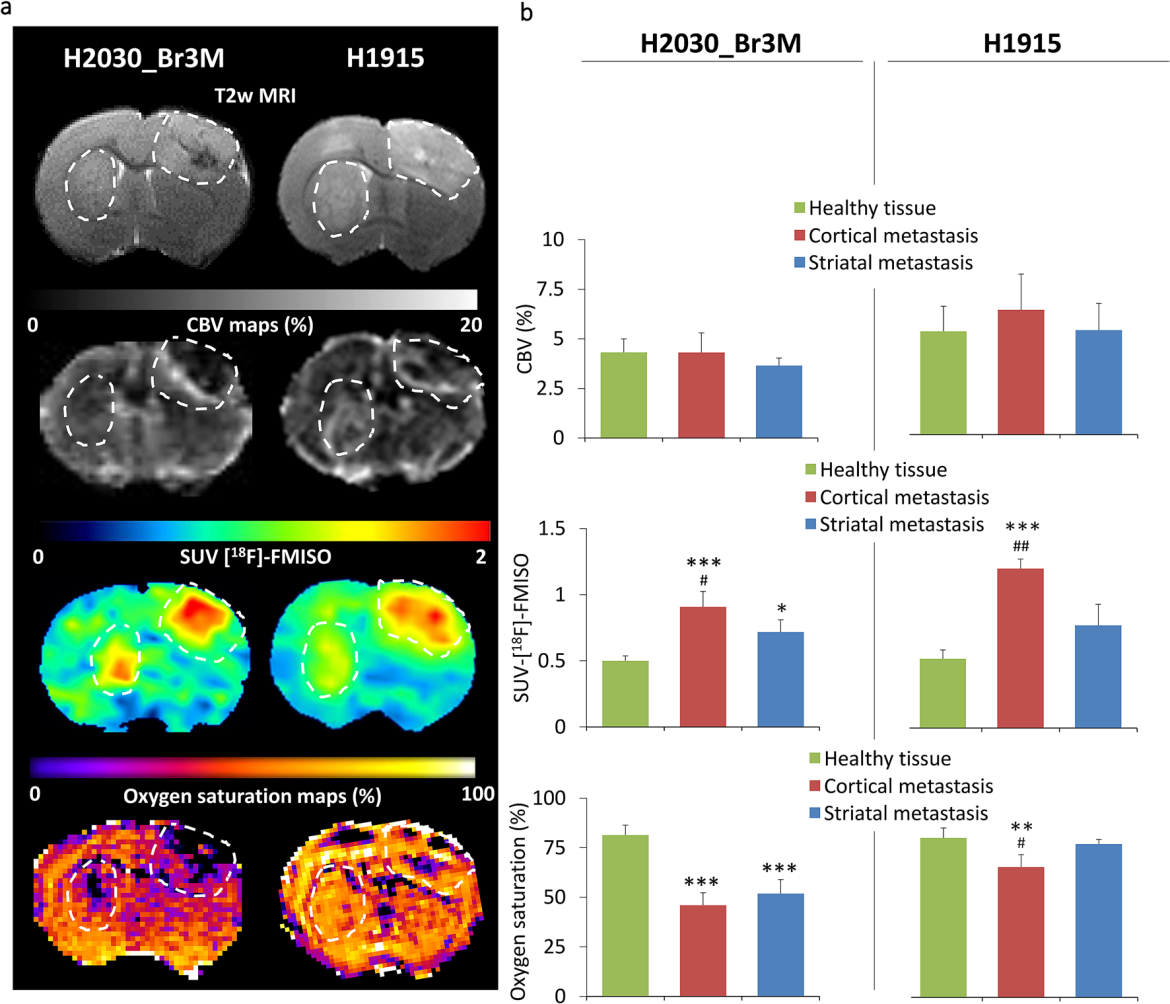
Multimodal imaging characterization of vascularization and hypoxia. (**a**) Representative images of H2030-Br3M and H1915 brain metastases (right and left respectively, hatched line). From the top to the bottom: T2w MRI, CBV-MRI map, [^18^F]-FMISO-PET and Oxygen saturation-MRI map. (**b**) Quantitative analyzes of multimodal imaging characterization. From top to the bottom: CBV (%), SUV-[^18^F]-FMISO and Oxygen saturation (%). Mean ± SD, n = 3 for H1915 and n = 4 for H2030-Br3M model, *p < 0.05, **p < 0.01 and ***p < 0.001 versus healthy tissue and ^#^*p* < 0.05 and ^##^*p* < 0.01 versus striatal metastasis.

The microenvironment of BM was further studied using multimodal imaging, an approach more adapted for future hypoxia-guided RT. Representative anatomical MRI for the H2030-Br3M and H1915 intracerebral models 20 days after inoculation are shown in Fig. 5a (top). As a related parameter of hypoxia, we firstly analyzed BM vascularization with MRI (CBV-MRI). For both models, MRI revealed similar CBV in cortical and striatal metastases as in healthy tissue. More interestingly, [^18^F]-FMISO-PET revealed pronounced hypoxia in the H2030-Br3M model in both regions (*p* < 0.05 and *p* < 0.001 vs. Control for striatal and cortical metastases and *p* < 0.05 between the two BM, Fig. 5b). For H1915, the heterogeneity in hypoxia observed by immunohistochemistry between the brain regions was confirmed by [^18^F]-FMISO-PET. The cortical metastases was more hypoxic than in the healthy tissue and also than in the striatal metastasis, which was not significantly different to healthy tissue (*p* < 0.001 vs. Control and *p* < 0.01 vs. striatal metastasis). SatO_2-_MRI was also used as another imaging readout of tumor hypoxia. SatO_2_-MRI revealed the presence of hypoxia and its spatial heterogeneity in the H1915 model (Fig. 5b; *p* < 0.001 and *p* < 0.05 for cortical metastases vs. Control and striatal metastasis, respectively).

These results showed, in BM preclinical models from human lung adenocarcinoma, the existence of hypoxia in BM microenvironment. These results support the concept that adapting treatment, especially RT, to hypoxia could improve tumor control. However, taking account of the marked intra-BM and inter-BM heterogeneity, robust tools are needed to map hypoxia. To this end, we have shown that [^18^F]-FMISO-PET and SatO_2_-MRI are of interest to characterize BM hypoxia and the inter-BM heterogeneity, and consequently may be useful for HIGRT.

### Added value of hypoxia image-guided radiotherapy (HIGRT) in preclinical models of BM

**Figure 6:**
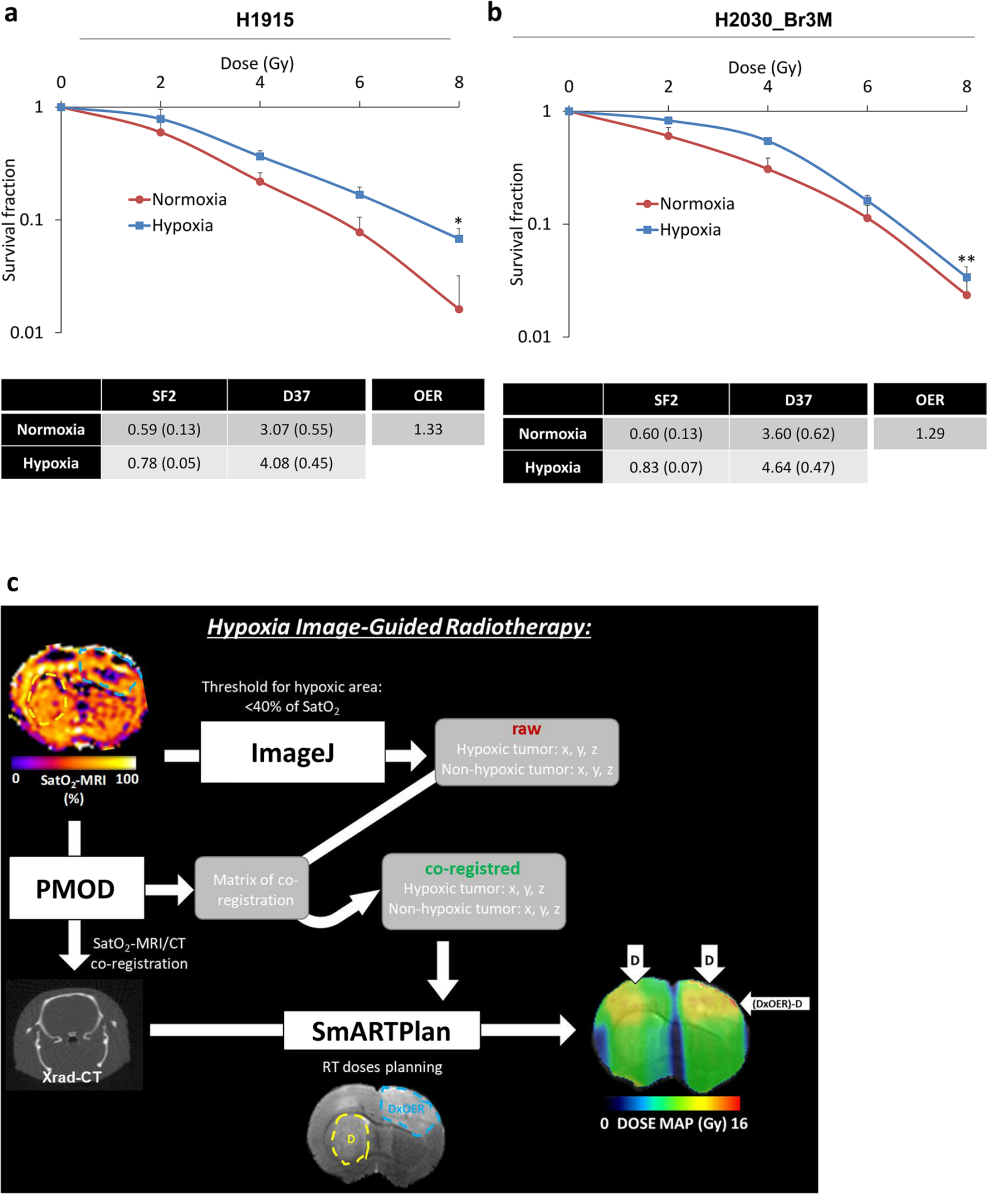
Radioresistance owed to hypoxia and HIGRT paradigm. (**a**) Quantitative analyzes of X-rays H1915 in vitro radiosensitivity in normoxia and hypoxia conditions (red and blue curves respectively). Mean ± SD, n = 4 for both conditions. *p < 0.05 for dose effect. SF2 = surviving fraction for 2 Gy; D50 = dose for which surviving fraction is 50%, OER (Oxygen Enhancement Ratio). (**b**) Quantitative analyzes of x-rays H2030-Br3M in vitro radiosensitivity in normoxia and hypoxia conditions (red and blue curves respectively). Mean ± SD, n = 4 for both conditions. *p < 0.05 for dose effect. SF2 = surviving fraction for 2 Gy; D50 = dose for which surviving fraction is 50%, OER (Oxygen Enhancement Ratio). (**c**) HIGRT paradigm showing the hypoxic and non-hypoxic BM revealed by SatO_2_-MRI leading to a boost of radiotherapy depending of the OER in the hypoxic BM.

The aim of this study was to evaluate the therapeutic potential of quantitatively adapting RT to hypoxia based on imaging for BM. As the intracerebral H1915 model presented the highest inter-BM heterogeneity in terms of hypoxia, the therapeutic potential of HIGRT was evaluated in this model. To address the quantitative boost of RT needed to counteract hypoxia-induced radioresistance, Oxygen Enhancement Ratio (OER) was calculated in vitro in this cell line. Clonogenic assay performed under normoxia and hypoxia (1% of O_2_) revealed an increase in survival fraction at 2 Gy (SF2) in hypoxia leading to an OER of 1.33 (*p* < 0.05, Fig. 6a). To further support the concept that radioresistance due to hypoxia is a hallmark of BM, a clonogenic assay was also performed on the H2030-Br3M cell line and similar results were obtained (Fig. 6b). As presented in Fig. 6c, SatO_2_-MRI was used to define a central hypoxic region to guide RT boost by a factor of 1.33 in hypoxic regions depending on the OER calculated in vitro. More details are presented in the “Methods” section.

**Figure 7:**
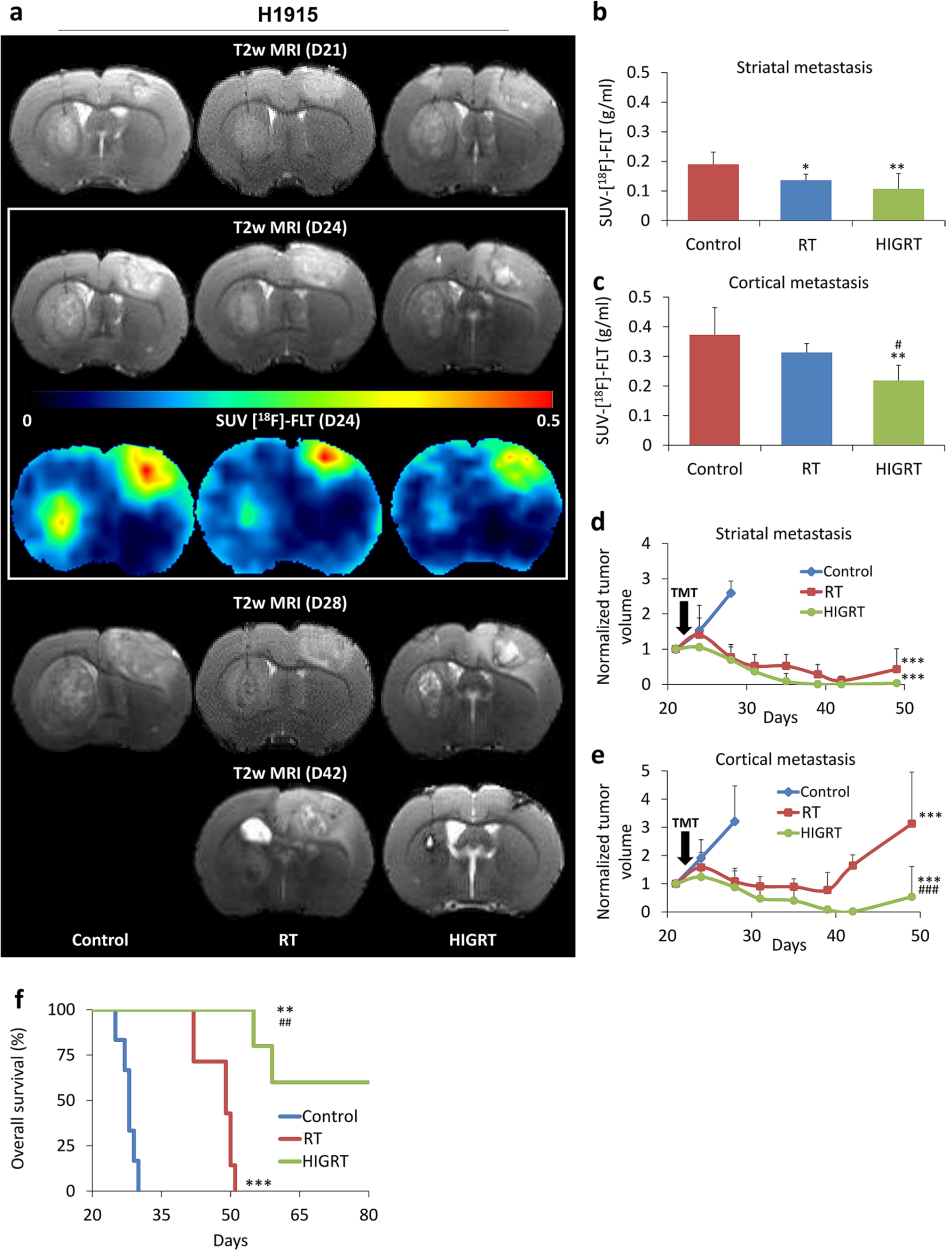
Treatment effect on tumor volumes, cell proliferation and overall survival. (**a**) Representative T2w-MRI and [^18^F]-FLT images for the three different groups of rats before and after treatments. From the top to the bottom: T2w-MRI before treatment (D21); T2w-MRI one day after treatments (D24); [^18^F]-FLT images at D24 and lastly, T2w-MRI at D28 and D42. Quantitative analyzes of SUV-[^18^F]-FLT one day after treatments in (**b**) striatal metastases and (**c**) cortical metastases. Mean ± SD, n = 7 for Control and RT groups and n = 6 for HIGRT group, *p < 0.05 and **p < 0.01 versus Control group and ^#^*p* < 0.05 versus RT group. Quantitative analyzes of tumor volume before and after treatment in (**d**) striatal metastases and (**e**) cortical metastases (TMT: treatments). Mean ± SD, n = 7 for Control and RT groups and n = 6 for HIGRT group, ***p < 0.001 versus Control group and ^###^*p* < 0.001 versus RT group. (**f**) Kaplan–Meier curves of survival, n = 6 for Control, n = 7 for RT and n = 5 for HIGRT groups. **p < 0.01 and ***p < 0.001 versus Control group and ^##^*p* < 0.01 versus RT group.

Prior to treatment of H1915 tumors in vivo at D21, tumor volumes in the different groups were similar (Fig. 7a, top). BM in the Control group showed continuous growth with a high cell proliferation evaluated with [^18^F]-FLT-PET at day D24 (Fig. 7a, from the top to the bottom and Fig. 7b–e). Following RT, tumor volume decreased in both cortical and striatal BM, however, as [^18^F]-FLT-PET suggests in Fig. 7a–c, this treatment was not able to control the hypoxic cortical BM, and a recurrence occurred only in these tumors (Fig. 7e). In contrast, in the non-hypoxic striatal metastasis, RT controlled tumor growth more effectively (Fig. 7d). The therapeutic effect of RT resulted in limited overall survival, although significantly greater than the Control group (Fig. 7f). HIGRT controlled both non-hypoxic and hypoxic BM and was supported by a significant decrease in cortical metastasis proliferation in comparison to Control and RT groups (*p* < 0.01 and *p* < 0.05 vs. Control and RT groups, respectively, Fig. 7c). Ultimately, HIGRT was able to control definitively three of the five (60%) non-hypoxic and hypoxic BM, with a significant increase in overall survival (*p* < 0.01, Fig. 7f). Cell proliferation immunostaining confirmed the [^18^F]-FLT-PET results, Sup Fig. 2a-c).

## Discussion

Current treatment options for patients with BM from lung cancer remains limited, firstly because BM are detected too late and standard external beam RT shows response heterogeneity between patients^21^. Secondly, RT may induce a decline in cognitive ability for long-term survivors^5^. Here we hypothesize that treatment’s response heterogeneity could be due to both inter-patient and inter-metastasis heterogeneity notably in terms of the microenvironment and, in particular, hypoxia^22^. Precise characterization of the BM microenvironment, including hypoxia could enable more personalized RT and, consequently, better tumor control.

In this study, we first investigated whether hypoxia is present in the microenvironment of BM from primary lung cancer at both clinical and preclinical levels. In the patient’s biopsies from non‑small cell lung cancer (NSCLC) BM, CA-IX staining was used as an endogenous molecular marker of hypoxia as it is the most used in clinical and research practices^23^. In patient studies, Proescholdt and colleagues have shown CA-IX expression in BM from lung, breast, kidney and melanoma cancer, and matched expression of CA-IX between primary lung cancer and BM was also found in another clinical study^24,25^. We have also further shown in the present manuscript, from four biopsies selected for their high or low CA-IX staining, concordance between both CA-IX and HIF-1α staining. Hypoxia was further characterized in different preclinical models of BM from lung cancers (H2030-Br3M, H1915; via intracerebral and intracardiac injections) using immunohistological staining (pimonidazole, HIF-1α) as well as imaging approaches. Subsequently, we assessed whether a therapeutic approach based on hypoxia imaging, HIGRT, has potential to counteract intra- and inter-BM heterogeneity. To reflect the clinical situation as closely as possible and to mimic the multiple foci observed in the clinical situation, we used preclinical models of BM in which human metastatic lung cancer cells were injected into two different brain regions (cortex and striatum).

Other studies have evaluated the presence of hypoxia in BM from lung cancer^8,9,26^. For example, Berghoff and colleagues have also shown, with HIF-1α staining, that hypoxia is a prognostic factor for overall survival^8,9^. These results highlight the importance of adapting RT according to the hypoxic status. Previous studies have shown the presence of hypoxia in BM preclinical models from primary breast cancer^27,28^ and none in preclinical models of BM from lung cancer. In this study, both intracerebral and intracardiac BM models of lung cancer showed hypoxic feature of BM. Of note, we have also obtained similar results from a breast cancer BM model induced by intracardial injection of human breast cancer cells (MDA-231 cell line) in nude mice. Indeed, two hundred thirty five BM from seven mice were analyzed and reported 69% positive pimonidazole staining (representative images of immunostained sections are presented in Sup Fig. 3; Sup Material).

Moreover, the preclinical existence of inter-BM microenvironment heterogeneity, in terms of hypoxia, according to the location (cortex vs. striatum) has not previously been reported. Here, we showed that the same number of cells injected at the same time in two different brain regions gave rise to tumors with similar BM volumes but different degrees of hypoxia in the H1915 and H2030-Br3M models. Interestingly, a preclinical study from Perera and colleagues showed, with intracardiac injection models, that BM preferentially developed in the cortex in comparison to deep brain^29^. Furthermore, two clinical studies have shown that BM more frequently occur in cortical brain than deep brain structures^30,31^. These data suggest that difference exist in the microenvironment of healthy cortex with other brain regions which could lead to an easier BM development in the cortex. Interestingly, we obtained preliminary results showing that acetyl-CoA content, a central metabolite^32^, is present at higher concentrations in healthy cortex compared to healthy striatum, a difference that is maintained in the presence of BM (Sup Fig. 4, Sup Material). These differences in metabolism/nutrients between the brain regions may differentially influence cancer cells, for example in term of proliferative rate, as underlined by the literature^33–36^ and also our results obtained by [^18^F]-FLT PET analyses (Sup Fig. 5, Sup Material). Indeed, the acetyl-CoA pool is the key metabolite to meet metabolic rewiring and the high bioenergetic demands of BM^37,38^. In addition, a clinical study has shown that acetyl-CoA metabolism is important with high level of acetyl-CoA oxidase 1 in BM from breast cancer in comparison to other metastatic sites, however differences between BM themselves was not investigated^39^.

The present work, together with those previously published^24,26,27,40–49^, highlight the importance of (i) characterizing the BM microenvironment in individual patients, and (ii) adapting external RT to the presence of hypoxia for better tumor control. Zakaria and colleagues have highlighted the importance of using more functional MRI biomarkers, such as diffusion-MRI, rather than conventional contrast-enhanced MRI for RT planning to improve BM control^50^. On the basis of our initial findings, we proposed that hypoxia imaging could enable external RT to be adapted to the tumor hypoxic environment (HIGRT). In this study, we showed that both [^18^F]-FMISO-PET and SatO_2_-MRI can detect hypoxia heterogeneity between cortical and striatal BM. SatO_2_-MRI for HIGRT, rather than [^18^F]-FMISO-PET, owing to the higher spatial resolution of MRI compared to PET, enable intra-metastasis hypoxia heterogeneity to be determined and guide precise HIGRT. In addition, the RT boost was based on OER calculation on in vitro experiments and was applied when the SatO_2_-MRI fell below a threshold from the literature (40%)^51^, resulting in a binary application of HIGRT. Other approaches have suggested to use non-linearity correlation between hypoxia imaging and real oxygen pressure in tissue (ptO_2_) for dose modulation in primary brain tumors^52,53^, however this approach has still to be validated at the preclinical and clinical levels. Integration into the clinic setting of functional imaging, like hypoxia imaging, in the RT workflow is still challenging. As we discussed in a previous review^54^, MRI based oxygenation biomarkers, such as SatO2 or Oxygen enhancement-MRI, would have the advantage of achieving high resolution in comparison to PET biomarkers, such as [^18^]-FMISO or [^18^]-FAZA. However, such MRI-based approaches would have to be considered as an indirect assessment of oxygen level, as they depend on the vascular compartment, whereas PET biomarkers are a direct assessment of cell hypoxia. From these imaging biomarkers, dose calculation can be computed either by contour using thresholds (as performed in this study), or by pixel. The latter is more dependent on the hypoxia imaging resolution, as well as the ability of the RT system to deliver multiple and small beams. In the clinical situation, inverse planning allows calculation of the optimal beams to reach a desired dose. Application of HIGRT for BM depends on whether the clinical center has a stereotactic radiosurgery system, which could enable delivery of a conventional dose in non-hypoxic tumor with a boost applied by dose painting according to contour in hypoxic BM. Dose painting by contour in a hypoxic BM would have the advantage, in comparison to whole tumor dose escalation, of being as close as possible to the hypoxic region and limiting non-useful dose deposition in both non-hypoxic tumor regions and nearby healthy brain tissue. However, as is done with the difference between the CTV and the PTV, a margin obtained from the hypoxia imaging could be added to the biological target volume (BTV) to take into account the uncertainties regarding clinical hypoxia imaging^55^.

In vitro analyses of OER is not possible to be undertaken in the clinic, however, this preclinical study aimed to understand more deeply the interest of HIGRT approach in terms of biological effect.

However, for the main part of this study, only two cell lines from metastatic human lung adenocarcinoma, were used (H1915 and H2030-Br3M) and further preclinical studies would be necessary to determine whether the presence of hypoxia and the therapeutic benefit of HIGRT are conserved in BM originating from other cancers such as breast and melanoma, the other major primary with a propensity to spread to the brain^21^.

## Conclusion

We show, at the clinical and preclinical levels that hypoxia is a hallmark of the microenvironment of BM from NSCLC lung cancer. BM heterogeneity in terms of hypoxia between patients and between metastases in preclinical models, highlights the potential interest of personalizing external RT to hypoxia to increase tumor control in comparison to conventional external RT.

## Methods

### Cell culture

H2030-Br3M adenocarcinomas of human origin that preferentially metastasizes to the brain and H1915 (ATCC, CRL5904) were used for this study. The cell lines were grown in supplemented DMEM (SIGMA-ALDRICH) at 37 °C in wet atmosphere.

### Rat brain metastases model

All animal investigations were performed under the current European directive (2010/63/EU) including ARRIVE guidelines. This study was undertaken in the housing and laboratories #F14118001 and with the permission of the regional committee on animal ethics (C2EA-54 CENOMEXA, projects #5065-#8941). Nude athymic rats (200-250 g, 8 weeks, female) were maintained in specific pathogen free housing*.* Rats were manipulated under general anesthesia (5% isoflurane for induction, 2% for maintenance in 70%N_2_O/30%O_2_). Body temperature was monitored and maintained at 37.5 ± 0.5 °C throughout the experiments. For the BM model, rats were placed in a stereotactic head holder and a scalp incision was performed along the sagittal suture. To investigate potential intermetastases hypoxia heterogeneity, two burr holes of diameter 1 mm was drilled in the skull, 3 and 3.7 mm lateral left and right respectively to the Bregma. H1915 and H2030-Br3M cells (5 × 10^4^ cells in 3 μl-PBS containing glutamine 2 mM) were injected over 6 min via a fine needle (30G) connected to a Hamilton syringe. The injection sites were the left caudate putamen at a depth of 6 mm and the right cortex at a depth of 2.5 mm. Animals were then followed by anatomical MRI over a 21 days period to follow BM development, then animals follow one of the two substudies detailed in Fig. 1a,b. Twenty-one days period between injection and treatment/imaging was chosen to allow measurable hypoxia with PET imaging owed to its spatial resolution. Methods for the intracardiac model of BM is detailed in Sup Material).

### Study 1: Characterization study of hypoxia in BM

#### Patients

Between May-2014 and May-2019, the brain tumor registry identified 28 patients aged over 18 years with a diagnosis of BM from primary NSCLC lung cancer. Overall, 28 patients (9 women/19 men) with a median age of 63.5 years were retrieved with sufficient tissue available for additional biomarker studies. All tumor specimens were reviewed by an experienced neuropathologist (FC): the pulmonary origin of BM was certified by the presence of a positive immunostaining for thyroid transcription factor-1 and cytokeratin 7 within the BM. As required by French laws, all patients provided informed consent, and the study was approved by the institutional ethics committee (North-West-Committee-for-Persons-Protection-III N°DC-2008–588). All methods involving humans were carried out in accordance with local guidelines and regulations and with the Declaration of Helsinki.

### Immunohistochemical characterization of hypoxia in BM biopsies

Hypoxia in BM biopsies was evaluated through the carbonic anhydrase-IX (CA-IX) expression, a recognized endogenous markers of hypoxia^20^ and HIF-1α. Slides were incubated with primary antibody against CA-IX (EPITOMICS, 1/100-30 min room temperature) and HIF-1α (Cell Signaling #36,169, 1/100-overnight at 4 °C) and revealed using the Novolink (LEICA) kit^56^. All slides were examined by one expert pathologist and positives or negatives CA-IX-BM were reported, which allows to obtain percentage of positives CA-IX-BM.

### Immunohistochemical characterization of hypoxia and vascularization in BM preclinical models

At the time of the last oxygenation and vascular MRI session (D22), immunohistochemical staining for rat-endothelial-cell-antigen (RECA-1), pimonidazole, CA-IX and HIF-1α were used as previously described^14^ to characterize vascularization and hypoxia, respectively. For the RT therapy study (study 2), 3 days after initiation of treatments (D24), immunohistochemistry was performed to assess tumor cell proliferation using Ki67.

### Preclinical magnetic resonance imaging (MRI)

For all MRI experiments, rats under anesthesia (5% isoflurane for induction, 2% for maintenance, in 70% nitrous oxide/30%oxygen) were placed in the prone position, their heads secured via ear and tooth bars. Respiration was monitored by a pressure-sensitive balloon around the abdomen. MRI was performed on a 7 Tesla magnet (Pharmascan, BRUKER, CYCERON biomedical imaging platform, Caen). A cross coil configuration was used (volume/surface coil, BRUKER, Ettlingen). After a scout imaging scan, the tumor-associated oedema was detected with a T2w sequence (RARE, acceleration factor of 8; TR/TEeff = 5000/62.5 ms; Number of EXperiments (NEX) = 1; 20 contiguous slices; resolution = 0.15 × 0.15x0.75 mm; Acq. time = 2 min). Then, cerebral blood volume and oxygen saturation were measured with Echo Planar Imaging (EPI). All EPI were acquired with a single shot, motion artefact and ghost free, double sampling k-space coverage with identical bandwidth and geometry (10 contiguous slices, resolution = 0.3 × 0.3x1.5 mm, excepted T2^2^*w which was acquired with a slice thickness of 0.3 mm for further correction of field inhomogeneities for the SatO2-MRI maps) with saturation slices at the edges of the field of view. Prior to the injection of contrast agents, five T2*w (TR = 20,000 ms, NEX = 3, 50 contiguous slices, resolution = 0.3 × 0.3x0.3 mm) and four T2w (TR = 20,000 ms, NEX = 3) EPI images were acquired with various echo times (TE for T2*w = 12, 15, 18, 21 and 24 ms and for T2w = 40, 60, 80 and 100 ms). Fractional Cerebral Blood Volume (fCBV): P904 (200 µmol/kg; Guerbet, France) was intravenously administrated and five minutes after, a last T2*w (TE = 12 ms) was acquired as previously described^57^.

### Positron emission tomography (PET)

[^18^F]-FMISO and [^18^F]-FLT were furnished by the LDM-TEP group (ISTCT laboratory, GIP CYCERON, Caen, France). Images were acquired by a preclinical PET SIEMENS Inveon preclinical system (CYCERON biomedical imaging platform, Caen). An X-ray scan was employed to generate attenuation maps just prior to an emission scan, lasting 20 min, initiated 120 min ([^18^F]-FMISO, 66 MBq/kg) and 40 min ([^18^F]-FLT, 66 MBq/kg) after radiotracer injection into the caudal vein as previously described ^58^. All images were reconstructed by the iterative OSEM-2D algorithm.

### Image processing and analyzes

Image analysis was performed with in-house macros based on the ImageJ software (http://rsb.info.nih.gov/ij/, 1997–2019). PET analyzes were performed by PMOD 3.1 (Pmod Technologies LLC).

#### MRI tumor volume

Tumor delineation was performed manually on all adjacent T2w slices. The tumor volume was achieved by multiplication of the sum of contiguous tumor surface areas with the slice thickness. The Region Of Interest (ROI) corresponding to the tumor, or to the healthy contralateral mirror ROI, was used thereafter for all other parameters.

#### Vascular and inflammation parameters

fCBV maps (expressed as a %) was calculated according to previous publications^59^. SatO_2_-MRI maps were computed from the equation published by Lemasson and collaborators ^51^.

#### MRI/PET co-registration

All MRI scans were executed such that the various MRI parameters were anatomically registered to each other. A first automatic registration (PMOD 3.1) was performed between T2w MRI (reference) and the X-ray scan (input) by normalized mutual information. Whenever necessary, the registration was manually refined. As a second step, PET parameters were co-registered to MRI parameters.

#### PET analyzes

ROIs defined on T2w MRI were transferred onto all PET images. To quantify [^18^F]-FMISO and [^18^F]-FLT uptakes, the measured tissue activity concentration (counts kBq/ml) was divided by the injected activity in kBq per gram of body weight (kBq/g) to give a Standardized Uptake Value (SUV, g/ml).

### Study 2: Effect of two distinct RT modalities (WBRT vs. HIGRT) on tumor control in preclinical BM model

As H1915 model exhibit inter-metastases heterogeneity in term of hypoxia, this model was chosen to conduct the image-guided RT study.

### Clonogenic survival assay

To characterize the radioresistance due to hypoxia in H1915 cells, a clonogenic assay was used in normoxic and hypoxic conditions^60^. For normoxic condition, BM cells were plated in 6-well plates (1500 cells/well), exposed to X-rays (0 Gy; 2 Gy; 4 Gy; 6 Gy and 8 Gy) 24 h after the cell seeding and kept in the incubator for a duration of 12 days. Then, colonies were stained with 2% crystal violet (Sigma-Aldrich) diluted in 20% ethanol. For hypoxic condition, cells were plated in 6-well plates (1500 cells/well) kept in an hypoxic chamber for 6 h (1% O_2_/5% CO_2_, 37 °C, Invivo2-500, RUSKINN, ABE) 18 h after cell seeding and then exposed to X-rays before keeping in the hypoxic chamber for 12 days.

A value of 1% O2 was chosen as it corresponds to a partial pressure of oxygen of 6.6 mmHg, which is close to previous reports of pO2 in brain tumors and lung cancer^53,61,62^. The effect of oxygen level in radiation efficacy was evaluate with quadruplicate experiment using, for each experiment, four wells per radiation dose. SF2, (survival fraction at 2 Gy), D37 (lethal dose corresponding to dose for SF = 37%) and OER (oxygen enhancement ratio) were obtained from the survival curves as previously described^63^.

### In vivo radiation treatments

After SatO_2_-MRI imaging session, at day 21, rats (n = 7 per group) were assigned in the three following treatment groups: Control, Whole Brain Radiation Therapy (WBRT) and Hypoxia Image-Guided Radiotherapy (HIGRT). These RT treatments were performed using a XRad-225Cx irradiator, (225 kV X-rays; 3.3 Gy/min, Precision X-Ray, Equipex Rec-Hadron, CYCERON biomedical imaging platform, Caen). A cone beam CT (CBCT) image was acquired using the XRad-225Cx irradiator for anatomical delimitation. Treatment planning and subsequent beam delivery were performed using SmART-Plan software (Precision X-Ray, USA and MAASTRO Clinic, Netherlands), with segmentation, targeting and planning performed using the CBCT image. For RT treatment, animals received panencephalic RT with a total dose of 12 Gy in 3 fractions of 4 Gy, using a 15 mm collimator under CBCT guidance. For HIGRT, SatO2-MRI was used to identify non-hypoxic and hypoxic tumor areas. Areas were considered hypoxic if the SatO2 level was below 40%^51^. SatO2-MRI was co-registered to the CBCT image from the irradiator, which allowed a co-registration matrix to be obtained and this was used to transfer non-hypoxic and hypoxic tumor localizations into the irradiator system geometry. Striatal normoxic BM received a 12 Gy dose using a 5 mm collimator. Hypoxic cortical metastases received 12 Gy with the 5 mm collimator, plus an additional dose of 4 Gy in the hypoxic area using a 2.5 mm collimator, to achieve a total dose of 12 Gy × OER. Based on the clonogenic assay, the OER was 1.33, which leads to a total dose in the hypoxic tumor area of 16 Gy (Fig. 6c).

### Survival study

After irradiation treatment, rats were followed for a survival study. Prior to the initiation of the study, we defined 80 days as an arbitrary endpoint for overall survival outcome or when total tumor volume overcome 150 mm^3^ or when tumor impair mobility.

### Statistical analyzes

All data are presented as mean ± SD. Student’s t-test was used to compare cortical and striatal metastases and one-way and two-way (group and time effects) ANOVA followed by Tukey’s post-hoc test was used to compare differences between treatment groups. A log-rank test was used to compare Kaplan–Meier curves. Statistical analyzes were obtained with JMP (SAS-Institute-Inc, USA).

### Ethics approval and consent to participate

As required by French laws, all patients provided informed consent, and the study was approved by the institutional ethics committee of Caen University Hospital (North-West Committee for Persons Protection III CPP N ° DC-2008–588 of Caen University Hospital, France), France. For animal experiment: all applicable international, national, and/or institutional guidelines (including the ARRIVE’s guidelines) for the care and use of animals were followed. All procedures performed in studies involving animals were in accordance with the ethical standards of the institution or practice at which the studies were conducted as detailed in the “Methods” section.

## Supplementary Information

Supplementary Legends.

Supplementary Information.

## Data Availability

The datasets used and/or analyzed during the current study are available from the corresponding author on reasonable request.

## Abbreviations

BM: Brain metastases
CA-IX: Carbonic anhydrase-IX
CBCT: Cone beam computed tomography
CBV: Cerebral blood volume
HIGRT: Hypoxia image-guided radiotherapy
MRI: Magnetic resonance imaging
OER: Oxygen enhancement ratio
PET: Positon emission tomography
PBS: Phosphate buffered saline
RECA-1: Rat endothelial cell antigen-1
SD: Standard deviation
SF: Survival fraction
RT: Whole brain radiotherapy
