# Supplementary material for: Multimodal evaluation of hypoxia in brain metastases of lung cancer and interest of hypoxia image-guided radiotherapy
Source: Sci Rep. 2021 May 27;11:11239. doi: 10.1038/s41598-021-90662-0 (PMC8159969; doi:10.1038/s41598-021-90662-0)
Supplement: Supplementary file 1 — Supplementary Legends. [file 41598_2021_90662_MOESM1_ESM.docx]

**Sup Fig.1: Immunohistochemical determination of carbonic anhydrase-IX (CA-IX) and HIF-1α expression in BM patients**

Representative images of CA-IX and HIF-1α staining (brown) with hematoxylin counterstaining (purple) on four BM biopsies from four patients with primary lung adenocarcinoma.

**Sup Fig.2:** **Immunohistochemical determination of treatment efficacy**

Representative images of Ki67 immunostaining (cell proliferation, red) with a Hoechst 33342 nuclear counterstaining (blue) for (**a**) cortical and (**b**) striatal H1915 metastases. (**c**) Quantification of the Ki67 immunostaining. Data represent the mean (SD) per group of the percentage of Ki67 positive cells analyzed with histology for H1915 cortical and striatal metastases. Mean ± SD, n=3, ***p<0.001 *vs* Control group and ^#^p<0.05 and ^###^p<0.001 *vs* RT group.

**Sup Fig.3: Pimonidazole detection by immunohistology in preclinical models of BM with intracardiac injection of human breast cancer**

Representative images of pimonidazole staining (red) with a Hoechst 33342 nuclear counterstaining (blue) on BM model from seven mice with human breast cancer (MDA-231-Br).

**Sup Fig.4: Acetyl-CoA concentrations in cortex and striatum from healthy tissue and with the presence of BM**

Quantitative analyzes of Acetyl-CoA concentration in cortex and striatal structures. Mean ± SD, n=3 for all groups except cortical metastasis (n=4), ***p<0.001 *vs* Healthy cortex, ^€€€^p<0.001 *vs* Healthy striatum, ^###^p<0.001 *vs* cortical metastasis and ^$$$^p<0.001 *vs* striatal metastasis.

**Sup Fig.5: Tumor cell proliferation in cortical and striatal metastases**

Representative images (left) and quantitative analyzes (right) of SUV-[^18^F]-FLT in H1915 brain metastases 24 days after tumor cells implantation. Mean ± SD, n=7, ***p<0.001 *vs* Striatal metastasis.

**Sup Fig.6:** **Dose deposition in BM and healthy brain tissues with WBRT or HIGRT**

(a) Quantitative analyses of dose deposition in healthy brain tissue and trachea as organ at risk in the two types of treatment. Mean ± SD, n=7 for RT and n=8 for HIGRT treatments, ***p<0.001 vs RT treatment. Dose-volume histogram, representing the dose distribution within a volume of interest in healthy brain tissue, trachea, striatal and cortical metastases for (b) WBRT and (c) HIGRT treatments.
