# Supplementary material for: Multimodal evaluation of hypoxia in brain metastases of lung cancer and interest of hypoxia image-guided radiotherapy
Source: Sci Rep. 2021 May 27;11:11239. doi: 10.1038/s41598-021-90662-0 (PMC8159969; doi:10.1038/s41598-021-90662-0)
Supplement: Supplementary file 2 — Supplementary Information. [file 41598_2021_90662_MOESM2_ESM.pptx]

## Slide 1
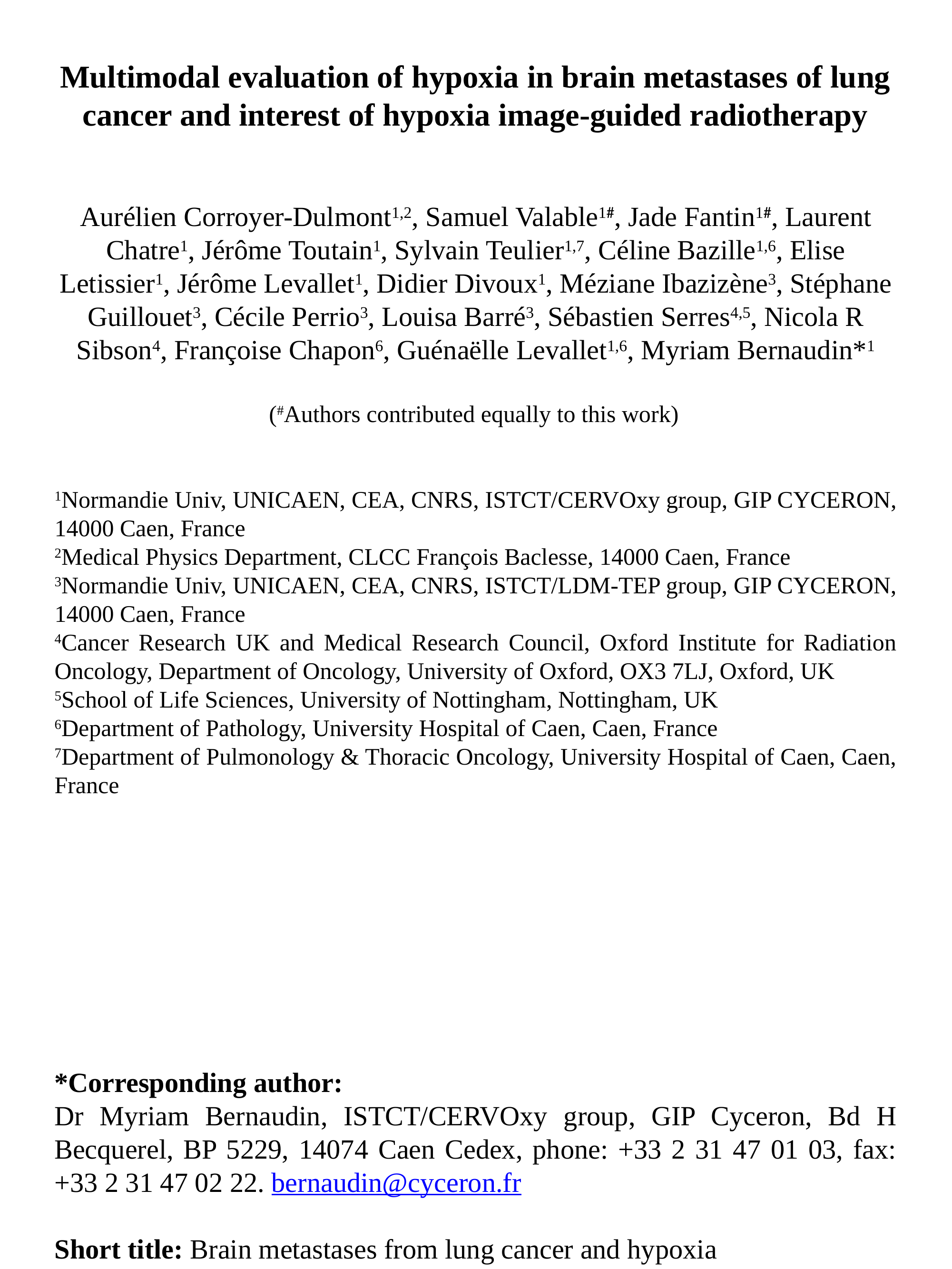

Multimodal evaluation of hypoxia in brain metastases of lung cancer and interest of hypoxia image-guided radiotherapy
Aurélien Corroyer-Dulmont1,2, Samuel Valable1#, Jade Fantin1#, Laurent Chatre1, Jérôme Toutain1, Sylvain Teulier1,7, Céline Bazille1,6, Elise Letissier1, Jérôme Levallet1, Didier Divoux1, Méziane Ibazizène3, Stéphane Guillouet3, Cécile Perrio3, Louisa Barré3, Sébastien Serres4,5, Nicola R Sibson4, Françoise Chapon6, Guénaëlle Levallet1,6, Myriam Bernaudin*1
(#Authors contributed equally to this work)
1Normandie Univ, UNICAEN, CEA, CNRS, ISTCT/CERVOxy group, GIP CYCERON, 14000 Caen, France
2Medical Physics Department, CLCC François Baclesse, 14000 Caen, France
3Normandie Univ, UNICAEN, CEA, CNRS, ISTCT/LDM-TEP group, GIP CYCERON, 14000 Caen, France
4Cancer Research UK and Medical Research Council, Oxford Institute for Radiation Oncology, Department of Oncology, University of Oxford, OX3 7LJ, Oxford, UK
5School of Life Sciences, University of Nottingham, Nottingham, UK
6Department of Pathology, University Hospital of Caen, Caen, France
7Department of Pulmonology & Thoracic Oncology, University Hospital of Caen, Caen, France
*Corresponding author:
Dr Myriam Bernaudin, ISTCT/CERVOxy group, GIP Cyceron, Bd H Becquerel, BP 5229, 14074 Caen Cedex, phone: +33 2 31 47 01 03, fax: +33 2 31 47 02 22. bernaudin@cyceron.fr
Short title: Brain metastases from lung cancer and hypoxia

## Slide 2
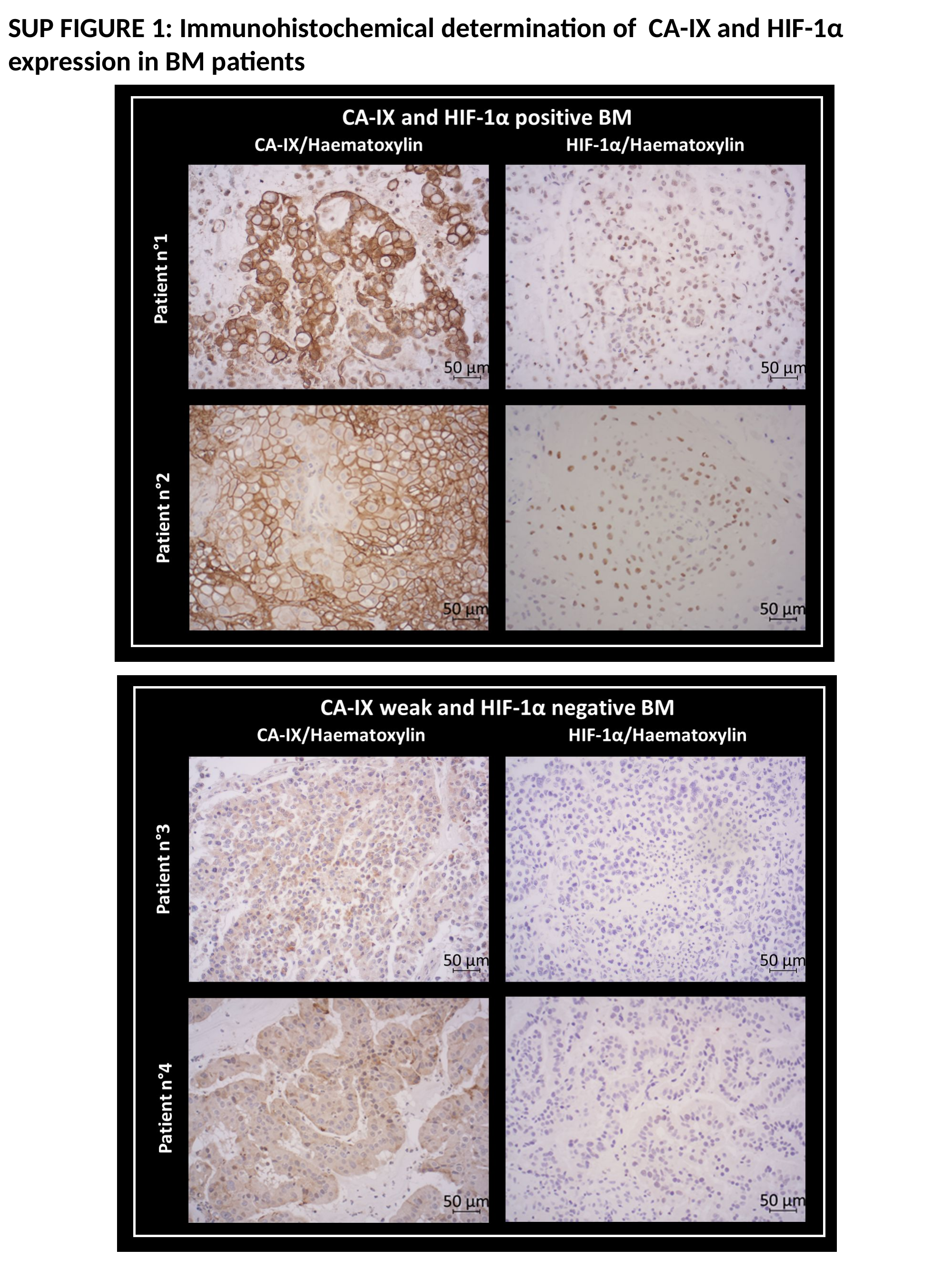

SUP FIGURE 1: Immunohistochemical determination of CA-IX and HIF-1α expression in BM patients

## Slide 3
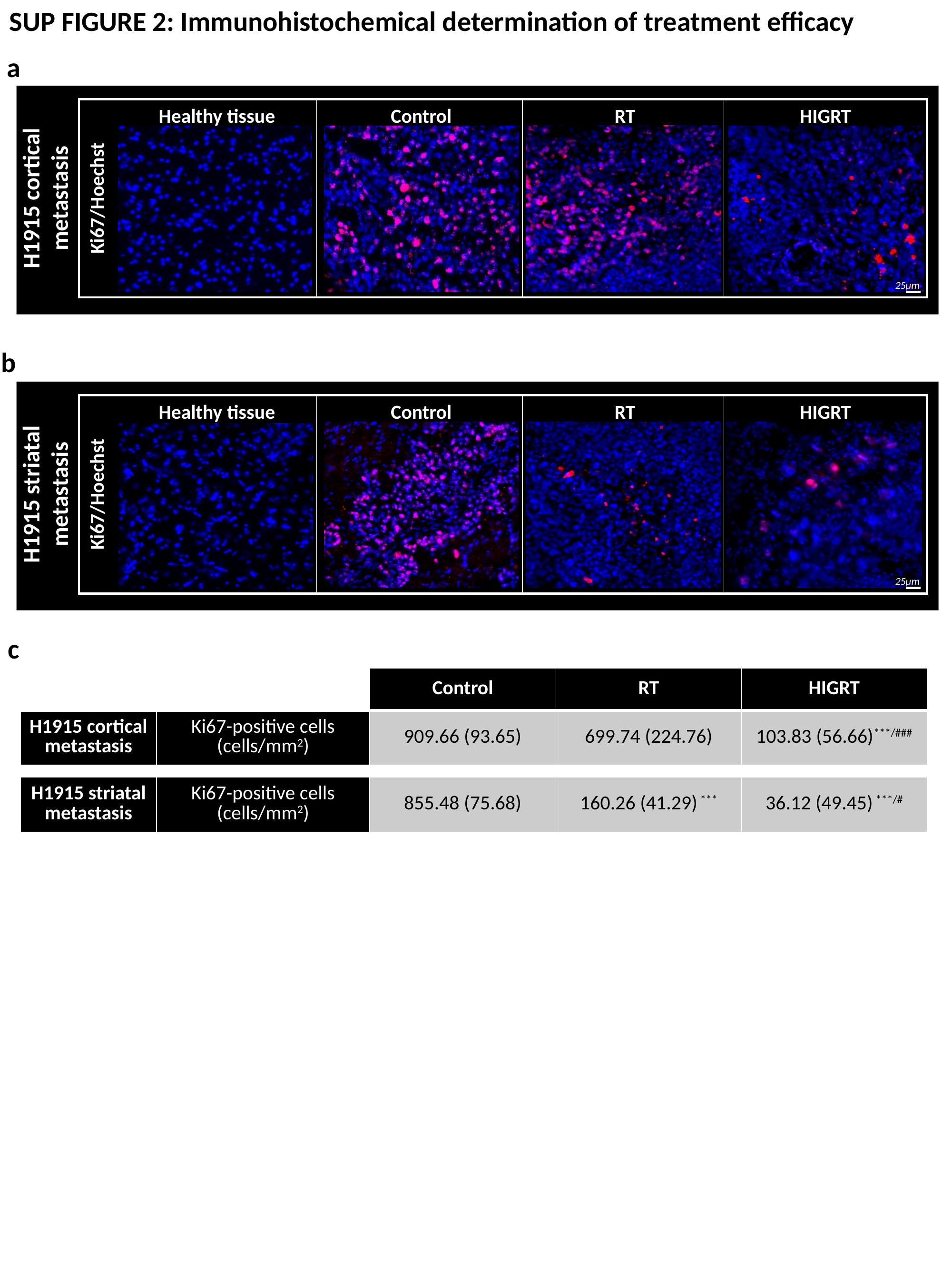

SUP FIGURE 2: Immunohistochemical determination of treatment efficacy
a
Healthy tissue
Control
RT
HIGRT
H1915 cortical metastasis
Ki67/Hoechst
25µm
b
Healthy tissue
Control
RT
HIGRT
H1915 striatal metastasis
Ki67/Hoechst
25µm
c
| | | Control | RT | HIGRT |
| --- | --- | --- | --- | --- |
| H1915 cortical metastasis | Ki67-positive cells (cells/mm2) | 909.66 (93.65) | 699.74 (224.76) | 103.83 (56.66)\*\*\*/### |
| | | | | |
| H1915 striatal metastasis | Ki67-positive cells (cells/mm2) | 855.48 (75.68) | 160.26 (41.29) \*\*\* | 36.12 (49.45) \*\*\*/# |

## Slide 4
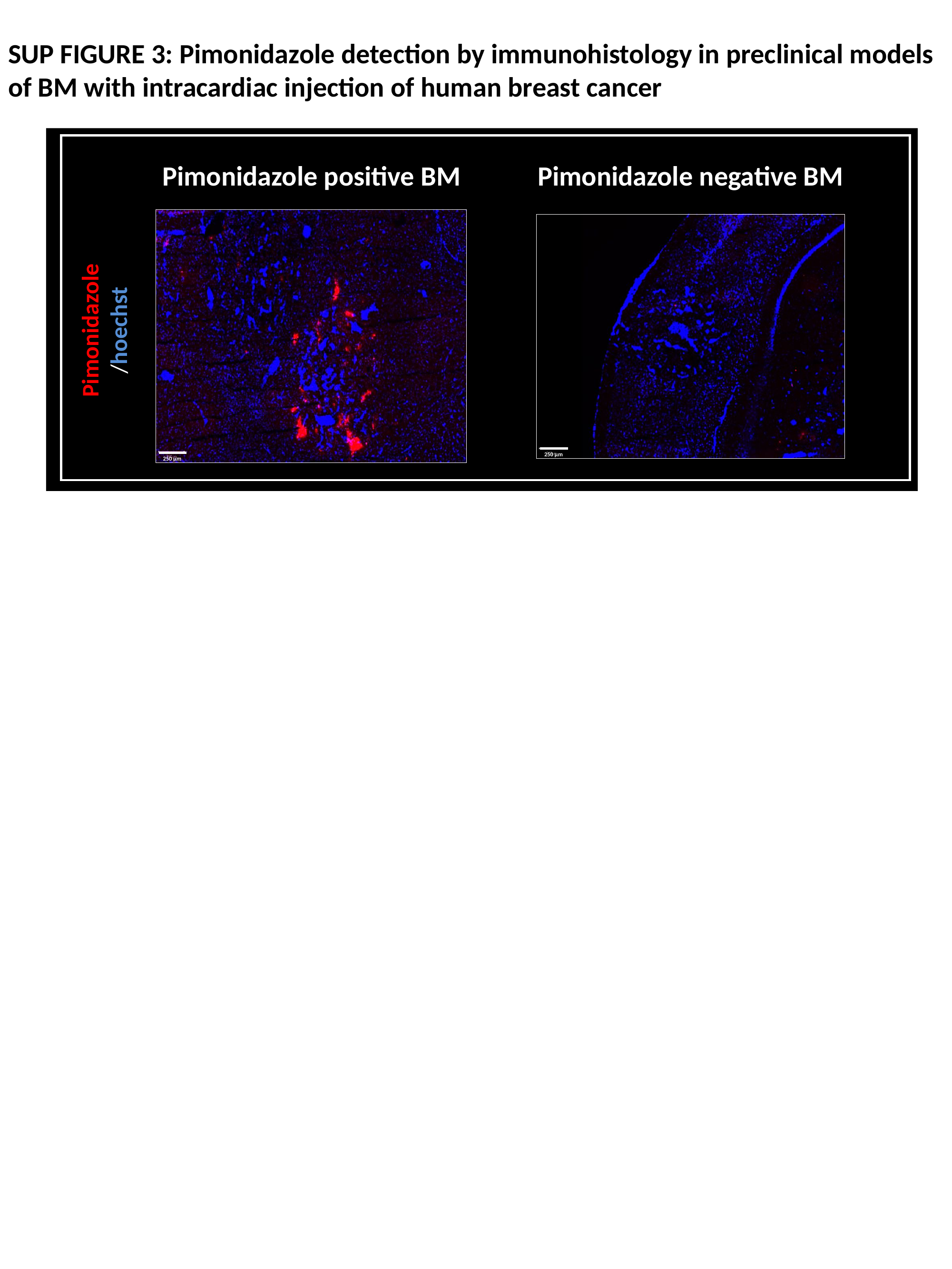

SUP FIGURE 3: Pimonidazole detection by immunohistology in preclinical models of BM with intracardiac injection of human breast cancer
Pimonidazole positive BM
Pimonidazole negative BM
Pimonidazole/hoechst
250 µm
250 µm

## Slide 5
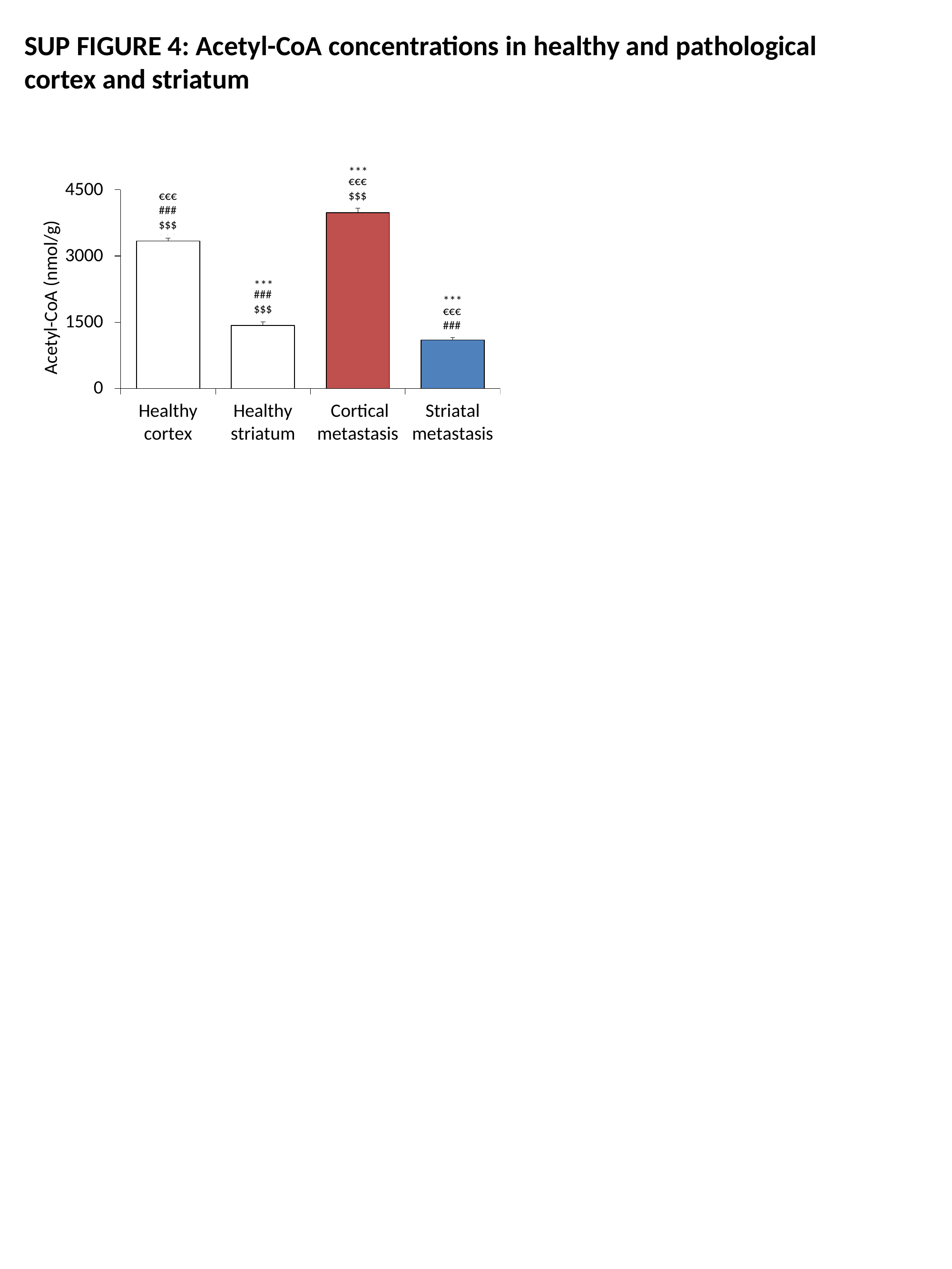

SUP FIGURE 4: Acetyl-CoA concentrations in healthy and pathological cortex and striatum
***
€€€
$$$
€€€
###
$$$
***
###
$$$
***
€€€
###

## Slide 6
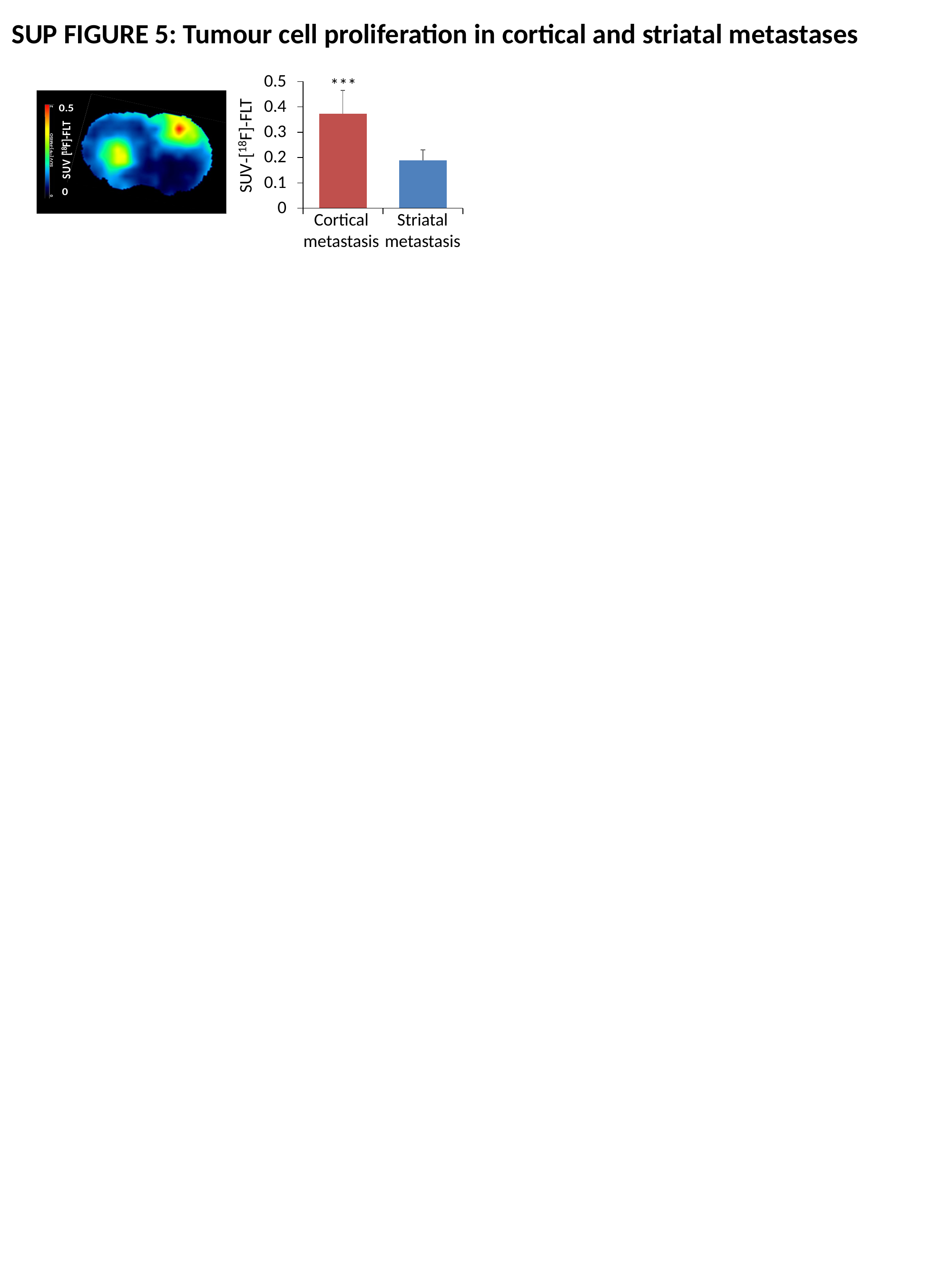

SUP FIGURE 5: Tumour cell proliferation in cortical and striatal metastases

## Slide 7
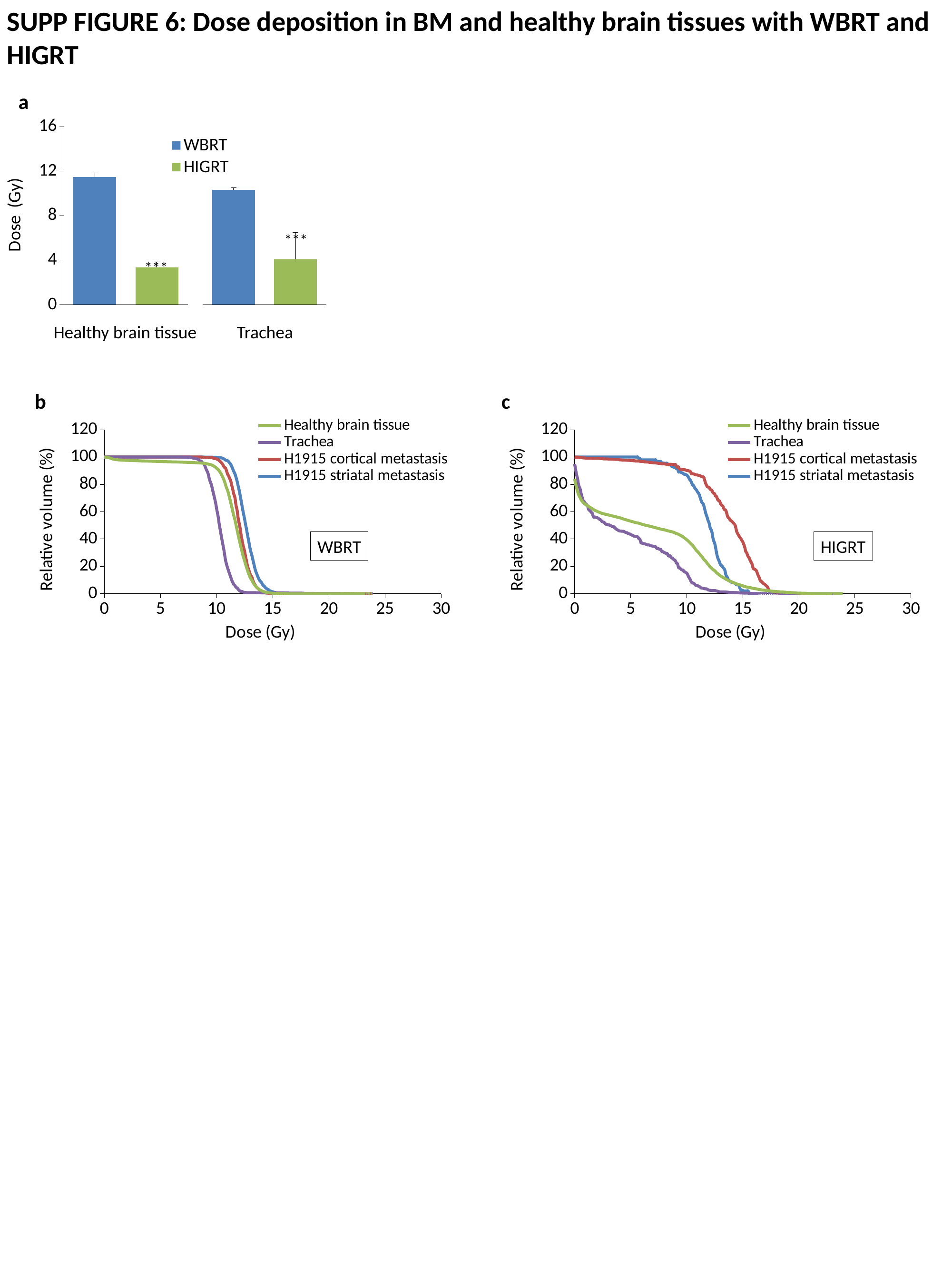

SUPP FIGURE 6: Dose deposition in BM and healthy brain tissues with WBRT and HIGRT
a
### Chart
| Category | dose reçue (en Gy) |
|---|---|
| Tissu cérébral sain | 10.34142857142857 |
### Chart
| Category | dose reçue (en Gy) |
|---|---|
| Tissu cérébral sain | 11.472857142857142 |WBRT
HIGRT
***
***
Healthy brain tissue
Trachea
b
c
### Chart
| Category | | | | |
|---|---|---|---|---|
### Chart
| Category | | | | |
|---|---|---|---|---|WBRT
HIGRT

## Slide 8
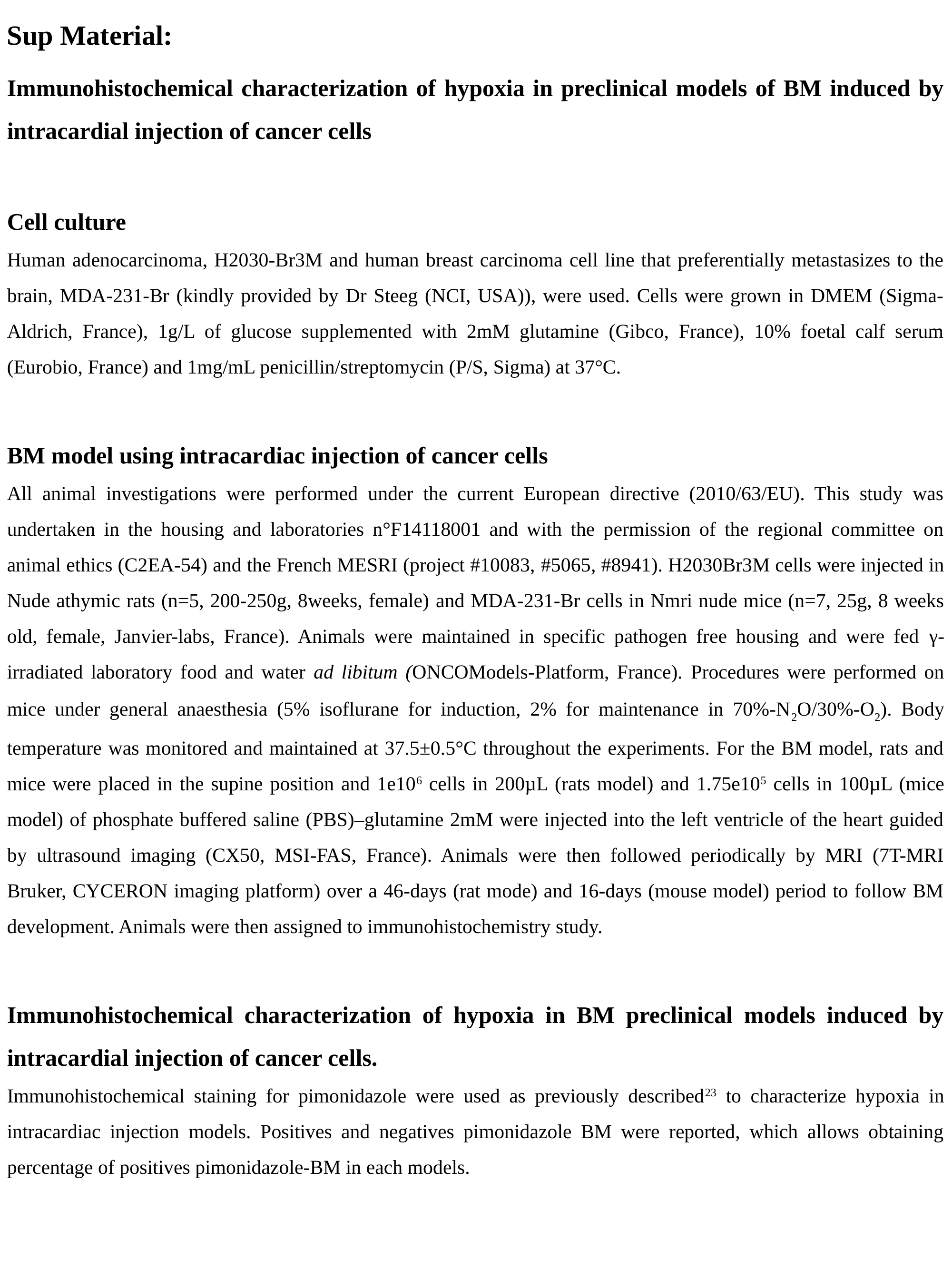

Sup Material:
Immunohistochemical characterization of hypoxia in preclinical models of BM induced by intracardial injection of cancer cells
Cell culture
Human adenocarcinoma, H2030-Br3M and human breast carcinoma cell line that preferentially metastasizes to the brain, MDA-231-Br (kindly provided by Dr Steeg (NCI, USA)), were used. Cells were grown in DMEM (Sigma-Aldrich, France), 1g/L of glucose supplemented with 2mM glutamine (Gibco, France), 10% foetal calf serum (Eurobio, France) and 1mg/mL penicillin/streptomycin (P/S, Sigma) at 37°C.
BM model using intracardiac injection of cancer cells
All animal investigations were performed under the current European directive (2010/63/EU). This study was undertaken in the housing and laboratories n°F14118001 and with the permission of the regional committee on animal ethics (C2EA-54) and the French MESRI (project #10083, #5065, #8941). H2030Br3M cells were injected in Nude athymic rats (n=5, 200-250g, 8weeks, female) and MDA-231-Br cells in Nmri nude mice (n=7, 25g, 8 weeks old, female, Janvier-labs, France). Animals were maintained in specific pathogen free housing and were fed γ-irradiated laboratory food and water ad libitum (ONCOModels-Platform, France). Procedures were performed on mice under general anaesthesia (5% isoflurane for induction, 2% for maintenance in 70%-N2O/30%-O2). Body temperature was monitored and maintained at 37.5±0.5°C throughout the experiments. For the BM model, rats and mice were placed in the supine position and 1e106 cells in 200µL (rats model) and 1.75e105 cells in 100µL (mice model) of phosphate buffered saline (PBS)–glutamine 2mM were injected into the left ventricle of the heart guided by ultrasound imaging (CX50, MSI-FAS, France). Animals were then followed periodically by MRI (7T-MRI Bruker, CYCERON imaging platform) over a 46-days (rat mode) and 16-days (mouse model) period to follow BM development. Animals were then assigned to immunohistochemistry study.
Immunohistochemical characterization of hypoxia in BM preclinical models induced by intracardial injection of cancer cells.
Immunohistochemical staining for pimonidazole were used as previously described23 to characterize hypoxia in intracardiac injection models. Positives and negatives pimonidazole BM were reported, which allows obtaining percentage of positives pimonidazole-BM in each models.

## Slide 9
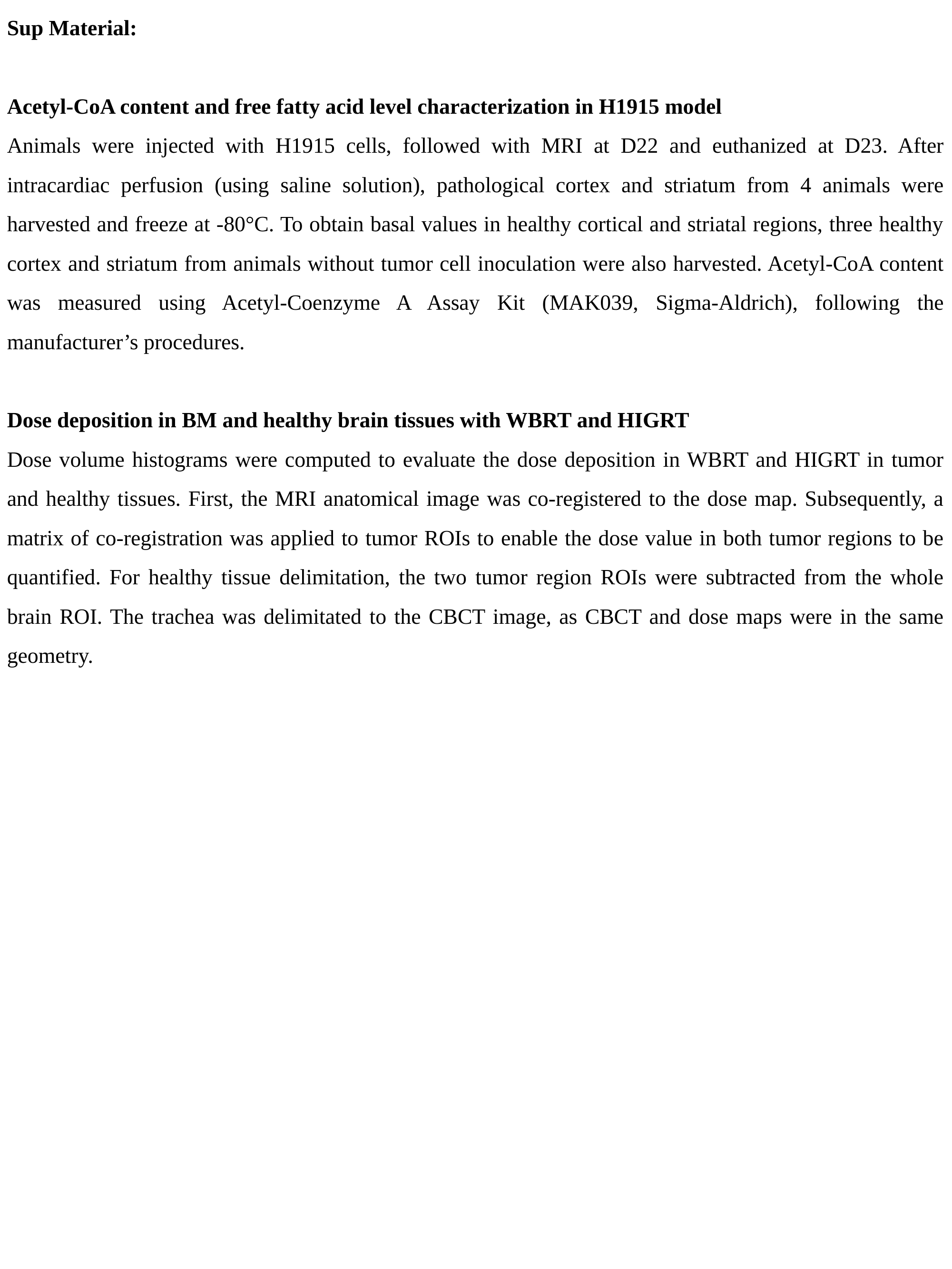

Sup Material:
Acetyl-CoA content and free fatty acid level characterization in H1915 model
Animals were injected with H1915 cells, followed with MRI at D22 and euthanized at D23. After intracardiac perfusion (using saline solution), pathological cortex and striatum from 4 animals were harvested and freeze at -80°C. To obtain basal values in healthy cortical and striatal regions, three healthy cortex and striatum from animals without tumor cell inoculation were also harvested. Acetyl-CoA content was measured using Acetyl-Coenzyme A Assay Kit (MAK039, Sigma-Aldrich), following the manufacturer’s procedures.
Dose deposition in BM and healthy brain tissues with WBRT and HIGRT
Dose volume histograms were computed to evaluate the dose deposition in WBRT and HIGRT in tumor and healthy tissues. First, the MRI anatomical image was co-registered to the dose map. Subsequently, a matrix of co-registration was applied to tumor ROIs to enable the dose value in both tumor regions to be quantified. For healthy tissue delimitation, the two tumor region ROIs were subtracted from the whole brain ROI. The trachea was delimitated to the CBCT image, as CBCT and dose maps were in the same geometry.
